# Regulated cell death and DAMPs as biomarkers and therapeutic targets in normothermic perfusion of transplant organs. Part 2: implementation strategies

**DOI:** 10.3389/frtra.2025.1575703

**Published:** 2025-04-24

**Authors:** Walter G. Land, Andreas Linkermann

**Affiliations:** ^1^ German Academy for Transplantation Medicine, Munich, Germany; ^2^ Laboratoire d'ImmunoRhumatologie Moléculaire, plateforme GENOMAX, INSERM UMR_S 1109, Faculté de Médecine, Fédération Hospitalo-Universitaire OMICARE, Fédération de Médecine Translationnelle de Strasbourg (FMTS), Institut Thématique Interdisciplinaire TRANSPLANTEX NG, Université de Strasbourg, Strasbourg, France; ^3^ Department of Integrated Medical Sciences, Medical Science Faculty, State University of Rio De Janeiro, Cabo Frio, Brazil; ^4^ Department of Medicine V, University Medical Centre Mannheim, University of Heidelberg, Mannheim, Germany; ^5^ Division of Nephrology, Department of Internal Medicine 3, University Hospital Carl Gustav Carus at the Technische Universität Dresden, Dresden, Germany; ^6^ Division of Nephrology, Department of Medicine, Albert Einstein College of Medicine, Bronx, NY, United States

**Keywords:** DAMP-induced alloimmunity, allotolerance, DCD, DBD, donor and recipient dendritic cells, allorecognition, normothermic regional perfusion, normothermic machine perfusion

## Abstract

This Part 2 of a bipartite review commences with the delineation of a conceptual model outlining the fundamental role of injury-induced regulated cell death (RCD) in the release of DAMPs that drive innate immune responses involved in early inflammation-related allograft dysfunction and alloimmune-mediated allograft rejection. In relation to this topic, the focus is on the divergent role of donor and recipient dendritic cells (DCs), which become immunogenic in the presence of DAMPs to regulate alloimmunity, but in the absence of DAMPs acquire tolerogenic properties to promote allotolerance. With respect to this scenario, proposals are then made for leveraging RCD and DAMPs as biomarkers during normothermic regional perfusion (NRP) and normothermic machine perfusion (NMP) of transplant organs from DCD donors, a strategy poised to significantly enhance current policies for assessing donor organ quality. The focus is then on the ambitious goal to target RCD and DAMPs therapeutically during NRP and NMP, aiming to profoundly suppress subsequently early allograft inflammation and alloimmunity in the recipient. This strategic approach seeks to prevent the activation of intragraft innate immune cells including DCs during donor organ reperfusion in the recipient, which is driven by ischemia/reperfusion injury-induced DAMPs. In this context, available inhibitors of various types of RCD, as well as scavengers and inhibitors of DAMPs are highlighted for their promising therapeutic potential in NRP and NMP settings, building on their proven efficacy in other experimental disease models. If successful, this kind of therapeutic intervention should also be considered for application to organs from DBD donors. Finally, drawing on current global insights into the critical role of RCD and DAMPs in driving innate inflammatory and (allo)immune responses, targeting their inhibition and/or prevention during normothermic perfusion of transplant organs from DCD donors - and potentially DBD donors - holds the transformative potential to not only alleviate transplant dysfunction and suppress allograft rejection but also foster allograft tolerance.

## 1 Background

This bipartite review highlights two seminal advancements in organ transplantation: the deepening conceptual understanding of injury-induced, DAMP-driven innate alloinflammatory and adaptive alloimmune responses and the growing adoption of normothermic organ perfusion preservation techniques by the transplant community.

Part 1 discussed – through the lens of the danger/injury model in Immunology - how the elicitation of innate alloimmunity in the recipient, along with alloantigen presentation, is driven by the DAMPs. Notably, two key categories of these danger molecules were distinguished: conventional DAMPs (cDAMPs), which are released from cells undergoing various types of regulated cell death (RCD) and inducible DAMPs (iDAMPs), which are secreted by cDAMP-activated cells of the innate immune system. As explained, cDAMPs released from types (or forms) of RCD - for simplicity sometimes referred to hereafter as *RCD*→*DAMPs* - accumulate within the donor organ during various critical periods of injury prior to the onset of innate alloimmune responses in the recipient after transplantation (1–5).

Such key periods of injury for a donor organ were identified and addressed, particularly the harmful events experienced by the donor before ICU admission (e.g., traumatic accidental events) as well as injuries to the donor organism associated with *donation after brain death* (DBD) and *donation after circulatory death (*DCD) conditions, further, insults during organ preservation procedures and, finally, injury sustained during postischemic reperfusion of the transplant in the recipient. As also emphasized in Part 1, these stages of injury are paralleled by an increasing immunogenicity of the donor organ that is conferred, within an inflammatory environment, by DAMP-activated dendritic cells (DCs) derived from both the donor and recipient. These highly professional cells among other antigen presenting cells (APCs) such as macrophages, monocytes, B cells, and endothelial cells play a key role in mediating direct, indirect, and semi-direct allorecognition.

Part 2 builds on the scenario presented in Part 1 by exploring the idea that if DAMPs released from cells undergoing RCD within the donor organ trigger an innate alloinflammatory and adaptive alloimmune response in the recipient, then inhibiting the emission of these DAMPs – and thereby preventing the activation of innate immune cells, including both donor and recipient DCs - could substantially minimize these responses. This strategy then may not only improve allograft dysfunction and reduce the risk of acute allograft rejection, but also potentially even foster allograft tolerance.

Accordingly, here, a strong rationale is presented for exploring DAMPs and RCD types as potential biomarkers and therapeutic targets in the context of normothermic regional perfusion (NRP) and normothermic machine perfusion (NMP) for advanced organ preservation. The focus is on the potential clinical benefits of these new diagnostic and therapeutic approaches, while considerations regarding the financial and logistical challenges of implementing NRP and NMP in organ transplantation are set aside from this discussion.

## 2 From donor organ injury-induced RCD to DAMP-orchestrated innate alloimmune responses: a concise synopsis of a conceptual model

The role of DAMPs in initiating, executing, and propagating sterile inflammatory responses is widely recognized and accepted (6, 7). Additionally, as discussed in Part 1, Sections 5.4.2 and 5.4.3, DAMPs play an indispensable role in promoting and shaping adaptive immune responses by driving the maturation of immature dendritic cells (iDCs) into immunostimulatory DCs. This maturation process, reflecting DC activation, provides upregulation of the essential costimulatory molecules, which are required to fully activate naïve T lymphocytes of the recipient in the transplantation setting. Over the past decade, a growing body of literature has detailed the release of DAMPs during solid organ transplantation. These studies have sought to elucidate the roles of specific DAMPs and their corresponding receptors in modulating the recipient immune response to the allograft (8–13).

Here, we concisely review the model of RCD→DAMP-driven trajectories involved in innate alloimmune-mediated acute allograft rejection, focusing on the role of DAMPs in the activation of donor- and recipient-derived DCs, which, via direct, indirect, and semi-direct allorecognition, initiate and amplify innate alloimmune responses. [Note, a detailed discussion of the distinctive role of DC subpopulations in this context is not addressed here, for detailed information, see (14, 15)].

### 2.1 Role of donor and recipient dendritic cells in alloimmunity and allotolerance

#### 2.1.1 Critical role of dendritic cells in controlling immunity

Of note, the immunogenic capacity of DCs was first observed within transplantation settings. However, Ralph Steinman suggested in 1978 (16) that DCs “*will prove to be a critical accessory cell required in the generation of many immune responses*”. Thereafter, DCs were soon recognized for their pivotal role in controlling immunity, including antitumor immunity (17, 18). Today, this scenario continues to be studied in both transplantation settings (19–22) and antitumor setups (23, 24). Not least inspired by the danger/injury hypothesis (1, 2), it has been suggested that DAMPs – or more specifically, a combination of RCD-associated DAMPs (i.e., cDAMPs and iDAMPs, see below) - control adaptive immunity, including antitumor immunity by driving “immunogenic” maturation of iDCs to immunostimulatory DCs (25). As activated “professionals” of APCs, they are now able to induce immunity by providing three signals: efficient presentation of processed antigenic peptides on the cell surface in the context of major histocompatibility complex (MHC) class I/class II to naïve T cells (signal 1), upregulation of costimulatory molecules (signal 2), and secretion of T cell-polarizing cytokines (signal 3) (5, 15, 26–28). The upregulation of costimulatory molecules is specifically attributed to the action of DAMPs.

However, inducing immunity is not the sole capacity of matured DCs, as they also have a divergent function in promoting immune tolerance to peripheral antigens - an emerging concept of “homeostatic maturation” of DCs (also termed “tolerogenic maturation” of DCs) under steady-state conditions that is garnering increasing attention (15, 28–31).

#### 2.1.2 Innate allorecognition in light of the danger/injury model

Innate allorecognition is a sophisticated immunological process critical in organ transplantation. It refers to the recognition of genetically disparate nonself MHC molecules and minor histocompatibility antigens of the donor by the recipient's T cells, triggering an alloimmune response. The precise mechanisms underlying innate allorecognition remain a topic of ongoing debate. One prevailing model proposes that monocytes and macrophages can directly recognize allogeneic nonself in the absence of inflammation and danger signals. This concept posits that innate allorecognition operates independently of lymphoid cells, inducing the maturation of APCs to initiate and sustain the adaptive alloimmune response (32). An alternative model, grounded in our danger/injury hypothesis, posits that innate alloimmune activation is triggered by DAMPs released from necrotic cells resulting from severe donor organ injury. These DAMPs promote the maturation of PRR-bearing iDCs into immunostimulatory DCs, thereby instigating and amplifying the alloimmune response. As this concept matches the core topic of this review, we will focus on this model.

As outlined in Part 1, Chaps. 4 and 5, a substantial accumulation of RCD→DAMPs occurs within a potential allograft throughout various injury-mediating periods in the donor, including (i) DBD or DCD conditions, (ii) preservation methods such as cold storage or normothermic perfusion preservation procedures, and (iii) ischemia/reperfusion injury (IRI) to the allograft in the recipient. The resulting excessive emission of DAMPs not only triggers a a robust alloinflammation within the donor organ but also – acting in tandem with donor-specific MHC/alloantigens – promotes the “immunogenic” maturation of iDCs to immunostimulatory DCs, which drive a powerful adaptive alloimmune response against donor-specific alloantigens (8–13, 20, 21) (also compare Part 1, Figure 7).

#### 2.1.3 Allorecognition: the three pathways to alloimmunity

**Figure 1:**
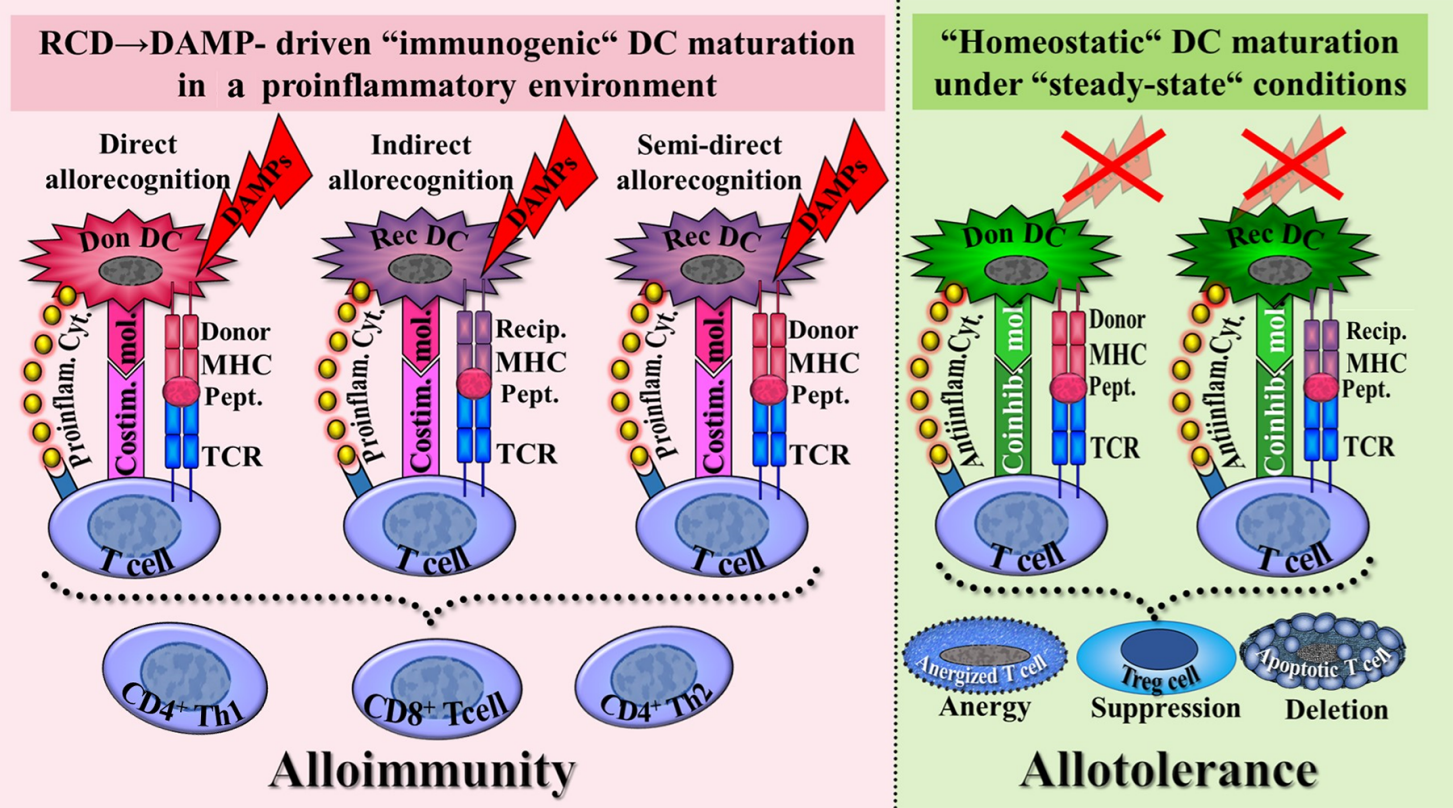
Schematic presentation of a conceptual model of dendritic cell (DC)-mediated pathways in T cell allorecognition as discussed for alloimmunity and allotolerance. *Alloimmunity*: In a proinflammatory environment (e.g., caused by ischemia/reperfusion injury), RCD→DAMP-activated donor-derived immunogenic mature DCs - via the direct pathway of allorecognition -, and RCD→DAMP-activated recipient-derived immunogenic mature DCs – via the indirect and semi-direct pathway of allorecognition – promote activation of recipient T cells, which drive an alloimmune response. The immunogenic pathways are characterized by (1) allopeptide presentation to the T cell receptor (TCR) by MHC (signal 1); (2) interaction of costimulatory molecules with their cognate receptors (signal 2); and (3) secretion of proinflammatory cytokines recognized by their cognate receptors of T cells (signal 3). *Allotolerance*: Under steady-state conditions, i.e., in the absence of injury-induced DAMPs, donor- and recipient homeostatic (tolerogenic) mature DCs interact with T cells resulting in T cell anergy, deletion, and conversion to regulatory T cells, all three mechanisms contributing to promotion of allograft tolerance. The homeostatic pathways are characterized by (1) allopeptide presentation to the T cell receptor by MHC (signal 1); (2) interaction of coinhibitory (immunomodulatory) molecules with their cognate receptors (signal 2); and (3) secretion of anti-inflammatory cytokines recognized by their cognate receptors of T cells (signal 3). compare also Figure 8 in Part 1. Antiinflam.Cyt., anti-inflammatory cytokines; Coinhib.mol., coinhibitory molecules; Costim. mol., costimulatory molecules; Don, donor; MHC, major histocompatibility complex; Pept, allopeptide; Proinflam. Cyt., proinflammatory cytokines; Rec, recipient; Recip., recipient; Treg, regulatory T cells.

The model scenario of alloantigen/DAMP-driven “immunogenic” DC maturation within the donor organ is instructive in deciphering how alloreactive naïve T cells residing in the recipients's secondary lymphoid organs are activated by recognition of (exogenous) alloantigens presented by DCs (Figure 1). Specifically, the model explains the facilitation of (i) recipient naïve alloreactive CD4^+^ and CD8^+^ T cells to recognize donor MHC class I and class II/alloantigens presented by DAMP-activated donor DCs (direct allorecognition), and (ii) recipient naïve CD4^+^ T cells to recognize recipient MHC class II/alloantigens presented by DAMP-activated recipient DCs (indirect allorecognition) (33–37). Noteworthy is here the phenomenon of cross-presentation: recipient naïve CD8^+^ T cells have been shown to recognize also MHC class I/exogenous peptides presented by DCs (38, 39). Moreover, recent studies demonstrate that alloreactive CD4^+^ T cells can also be activated through the recognition of donor MHC/alloantigens expressed on the recipient DCs, a process referred to as semi-direct allorecognition (also called MHC cross-dressing, Figure 1). This phenomenon occurs via the transfer of MHC/alloantigens through cell-to-cell contact or extracellular vesicles, following the semi-direct pathway (36, 37). A few additional details are provided in the subsequent sections.

##### 2.1.3.1 Donor DC-mediated direct allorecognition

In particular, the generation of RCD→DAMPs on the donor side highlights the significant impact of donor-derived DC-mediated direct allorecognition (40), which appears to be more responsible for acute rejection. This effect is notably substantial because alloantigen-presenting donor-derived DCs are activated by DAMPs during multiple periods of injury to the donor organ, that is, before its implantation into the recipient, but also again during IRI after transplantation (34, 35). In fact, this is the reason for having described in Part 1, Chap. 4 more carefully the various scenarios in the deceased donor leading to the emission of RCD-induced DAMPs in donor organs.

##### 2.1.3.2 Recipient DC-mediated indirect and semi-direct allorecognition

By contrast, DAMP-activated recipient DCs presenting allopeptides bound to their self-MHC molecules mediate indirect allorecognition, which involves the release of RCD→DAMPs during IRI to the donor organ in the recipient. This type of allorecognition is thought to be much longer lasting and mediate the progression of subacute/chronic rejection [compare also (34, 35)].

Additionally, the process of semi-direct allorecognition is gaining increasing attention (36, 37). In fact, allogeneic MHC class I and class II molecules were shown to be regularly transferred from donor cells to recipient APCs after allotransplantation. Emerging evidence suggests that this semi-direct alloantigen presentation by recipient DCs – rather than direct allorecognition - may play a central role in activating alloreactive T cells against intact donor MHC class I/class II molecules, thereby driving the early alloimmune response that leads to acute allograft rejection (36, 37).

#### 2.1.4 Allorecognition under steady-state condition: the way to allotolerance

In addition to their critical role as the initiators of adaptive immunity, DCs in their function as tolerogenic DCs [tolDCs, also termed regulatory DCs (DCregs)] are also involved in the induction and maintenance of central and peripheral immune tolerance. In the light of the concept of “homeostatic maturation”, under peripheral steady-state conditions, these homeostatically matured DCs present self antigens to self-reactive T cells that have escaped thymic selection. Such DCs are distinguished by their low expression of cell surface MHC gene products and costimulatory molecules, but high expression of coinhibitory molecules (also denoted as immunoregulatory or immunomodulatory molecules), and secretion of anti-inflammatory cytokines. Consistent with the maintenance of homeostasis, these homeostatically matured DCs interact with T cells in the steady state resulting in peripherally induced mechanisms of tolerance including T cell clonal anergy, deletion (apoptosis), and conversion to peripheral regulatory T cells [pTregs, Figure 1, for further reading, see (21, 30, 31, 41–43)]. Evidently, this concept is fundamentally aligned with the danger/injury model, which posits that the presentation of peripheral antigens, including nonself antigens such as alloantigens, by DCs under healthy steady-state conditions - marked by the absence of (i) DAMPs, (ii) costimulatory signals, and (iii) their associated specific inflammatory processes – does not inherently induce immunity. Instead, it promotes immune tolerance, including allotolerance (44–47).

Given their outstanding capacity to induce T cell tolerance, tolDCs are increasingly recognized as potential key mediators in the induction of allograft tolerance. Indeed, there is considerable interest in the potential of tolDC therapy to promote transplant tolerance. Consequently, DCs have been applied as cell-based immunotherapy in early phase I/II clinical trials in organ transplantation [for further reading, see Thomsen et al. (48), Ochando et al. (42), and Que et al. (49)].

The quest for induction of allograft tolerance is ongoing (45, 50). Within the framework of the danger/injury model, achieving the goal of “homeostatic DC maturation” to promote allograft tolerance requires preventing DAMP-induced “immunogenic DC maturation.” This objective necessitates ensuring the allograft is entirely devoid of DAMPs at the time of implantation, thereby establishing an intragraft steady state.

#### 2.1.5 Action of tolerogenic DCs as the goal of therapeutic interventions

Overall, over the past 4 decades, an intricate interplay between (i) RCD-associated DAMPs, (ii) activated immunostimulatory DCs in their role as key mediators of allorecognition, and (iii) the subsequent generation of adaptive alloimmune responses has been analyzed and documented. The interpretation of these findings in the context of alloimmune-mediated allograft rejection, as it is currently being discussed, is pragmatically and briefly outlined in the following section.

In parallel, experimental and clinicals studies have been designed and conducted to explore the potential of leveraging the tolerogenic properties of tolDCs to promote allograft tolerance through application of various tolDC-based therapeutic strategies. The discussion below on RCD and DAMPs as therapeutic targets will focus on how therapeutic interventions applied during normothermic perfusion of transplant organs may contribute to achieving this tolDC-centered goal of successful allotolerance induction.

### 2.2 *Regulated cell death*→*DAMP* - driven innate alloimmune pathways resulting in allograft rejection

**Figure 2:**
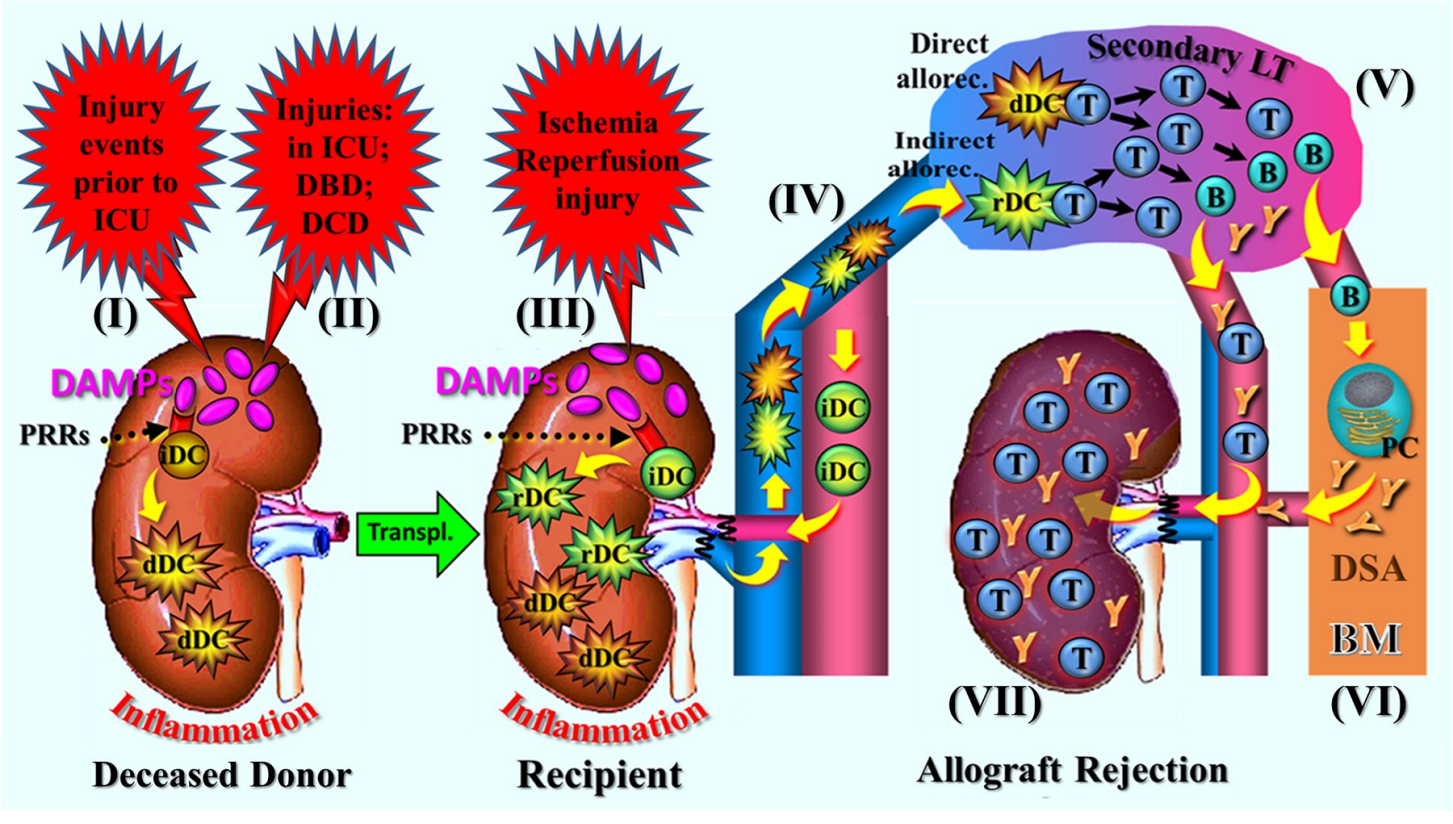
Schematic presentation of a narrative synopsis model of injury-induced, RCD→DAMP-driven innate alloimmune responses resulting in allograft rejection, exemplified by various injuries to the donor organ prior to its implantation and subsequent postischemic reperfusion-mediated oxidative injury after implantation into the recipient. The figure shows sequelae of steps of molecular and cellular trajectories as described. **(I)** Injury events (traumatic, cerebro-cardiovascular) to the donor/donor organs prior to admission to the ICU and **(II)** injuries during the stay in the ICU and under DBD or DCD conditions. The injurious events cause RCD (not shown) in potential transplant organs that is associated with release of DAMP, which activate donor PRR-bearing innate immune cells including immature DCs (iDCs). This leads to creation of an inflammatory milieu and immunogenic maturation of donor-specific iDCs into immunostimulatory DCs. **(III)** Following donor organ transplantation, postischemic reperfusion injury-induced DAMPs (released from RCD, not shown) re-trigger an inflammatory response; in addition, they promote immunogenic maturation of graft-infiltrating recipient-derived iDCs into immunostimulatory DCs. **(IV)** Emigration of donor and recipient DCs into secondary lymphatic organs of the recipient, followed by interaction of these cells with naïve T cell: that is, the phenomena of direct and indirect allorecognition (semi-direct allorecognition not shown). **(V)** Initiation of the adaptive alloimmune response with massive proliferation of T and B cells and alloantibody-secreting plasma cells. **(VI)** Long-lived plasma cells finding their niche in the bone marrow to continue production of donor-specific antibodies. **(VII)** Infiltration of the allograft by cytotoxic T cells and donor-specific antibodies leading, when untreated, to rejection. for didactic reasons, the renal artery is drawn ventral to the vena cava (anatomically it is dorsal); also note: for details of the various injuries to the donor organ, see Part 1, Chap. 4 and Chap. 5. allorec., allorecognition; B, B cell; BM, bone marrow; dDC, donor-derived dendritic cell; DBD, donation after brain death; DCD, donation after circulatory death; DSA, donor-specific antibodies; LT, lymphoid tissue; PC, plasma cell; PRRs, pattern recognition receptors; T, T cell; Transpl., transplantation.

As noted earlier, the cellular and molecular trajectories that ultimately lead to allograft rejection are triggered by cDAMPs, which are released by cells undergoing RCD due to various periodically occurring injuries to the donor (Figure 2). Notably, the sources of DAMPs operating within potential transplant organs vary significantly between DBD and DCD conditions: In DBD, RCD→DAMPs derive from both brain injury and peripheral tissue disturbances, whereas in DCD the sources are not well defined, but they may primarily originate from ventilator-induced lung injury and/or (repeated) systemic IRI caused by cardiac arrest/resuscitation (for more details, see Part 1, Sections 4.2.3 and 4.3.2). By contrast, the source of the DAMPs in IRI is reliably defined as the oxidative injury that is localized to the allograft (Part 1, Chap. 5).

During each of these injury-conferring periods, RCD→DAMPs reach the donor organ, where they are recognized by PRR-bearing cells of the donor's innate immune system, promoting an autoinflammatory response. In parallel, RCD→DAMPs activate PRR-bearing donor-specific iDCs, prompting their transformation into immunostimulatory DCs. After removal of transplant organs, additional DAMPs may be generated in the course of organ preservation procedures.

Following transplantation, IRI to the donor organ induces renewed damage in terms of an oxidative injury, which is again accompanied by the emission of RCD-associated DAMPs and the creation of an alloinflammatory environment. Additionally, RCD→DAMPs activate residual donor-derived iDCs as well as recipient-derived iDCs that have migrated into the donor organ and taken up donor alloantigens, transforming them into immunostimulatory DCs. Likewise, recipient-derived DCs may get activated that express donor MHC/alloantigens (Figure 2). Once activated, both donor- and recipient-derived immunostimulatory DCs migrate from the peripheral donor organ into the recipient's lymphoid tissue, such as lymph nodes and the spleen, where they interact with naïve alloreactive T cells.

This sequence of cellular and molecular signaling pathways illustrates the process of the abovementioned direct, indirect and semi-direct allorecognition, which is followed by the typical efferent stages of an adaptive alloimmune response, characterized by extensive T and B cell differentiation and proliferation, and by donor-specific alloantibody production that ultimately results in acute allograft rejection [Figure 2, for details of further CD4^+^ and CD8^+^ T cell differentiation into effector cells as well as B cells and alloantibodies, see recent reviews in (51, 52)].

### 2.3 Outlook

As is common with a brief depiction of new advances in scientific research, the presentation of this conceptual model of *RCD*→*DAMP*-driven trajectories involved in innate alloimmune-mediated acute allograft rejection is incomplete. Indeed, the immunological processes governing allograft destruction and survival are highly complex, involving not only various DC populations but also a diverse array of effector and regulatory cell types. Considering this intricate interplay, utilizing normothermic perfusion techniques as a platform to induce allograft tolerance represents a bold and ambitious approach. Nevertheless, it is a concept worthy of exploration.

## 3 Advancements in normothermic perfusion of DCD donor organs: enhancing viability assessment and enabling pretransplant therapeutic interventions

Since the 1970s, transplant surgeons relied solely on static cold storage (SCS) to ensure adequate preservation of retrieved organs, accepting certain degrees of ischemia-related organ dysfunction as a consequence. In recent times, however, the increasing shortage of suitable transplants has required the acceptance of organs from DCD donors but also marginal donors, such as expanded criteria donors (ECD), a policy that has led to the development and introduction of dynamic perfusion strategies. Indeed, depending on criteria such as the chosen temperature, the perfusion solution, supplemental oxygen supply, and timing of perfusion, a variety of approaches to organ perfusion has been published. In general, all these approaches have contributed to enhancing preservation-associated, ischemia-caused organ dysfunction, though they showed variable degrees of success in improving outcomes. However, NRP and NMP have recently moved to the forefront of attention. These two new normothermic perfusion techniques, which are currently mainly applied to organs from DCD donors, guarantee that the organs are permanently perfused with oxygenated blood or oxygen carrier, medications, and nutrients at body temperature during storage. The advantage here is that normal cellular metabolism as a fundamental principle of biology is restored with recovery of cellular energy status [replenishment of adenosine triphosphate (ATP) synthesis].

This methodology not only enhances conventional preservation procedures but also offers the possibility of assessing donor organ function under near-physiological conditions, allowing real-time viability assessment and repair of deceased-donor organs prior to transplantation. Additionally, they enable targeted therapeutic interventions to be performed. The following sections, outlined by authors outside this specialized field, offer a brief synopsis of the two normothermic perfusion techniques, highlighting their advantages in providing a platform for organ viability and function assessment, as well as enabling therapeutic interventions prior to transplantation.

### 3.1 *In situ* normothermic regional perfusion before organ retrieval

*In situ* NRP prior to explantation, originally developed in Barcelona, Spain for recovering organs from uncontrolled DCD donors has evolved as the favored method for improving the outcomes from organs obtained from controlled DCD and uncontrolled DCD donors (53–57) (for uncontrolled and controlled DCD, see Part 1, Section 4.3 and Table 1). The concept of this approach is grounded in an effort to tackle the heightened risks of complications associated with the detrimental effects of donor warm ischemia time, including primary nonfunction and biliary complications in liver transplantation, primary nonfunction and delayed graft function in kidney transplantation, venous thrombosis in pancreas transplantation, and high incidence of mechanical support in heart transplantation.

In contrast to rapid recovery after conventional *in situ* cold preservation, NRP in DCD minimizes the ischemic damage caused by the prolonged warm ischemia time by restoring circulation after death is declared and before the organ are flushed with cold preservation solutions during organ procurement. In executing this maneuver, the perfusion can be restricted to the abdomen or abdomen and chest [for further reading, see (53, 54)].

The technical aspects of NRP have been comprehensively reviewed by Watson and colleagues (58). In brief: NRP entails restoring blood circulation to the abdominal (and thoracic) organs while preventing brain perfusion. This is accomplished using a modified extracorporeal oxygenation circuit (ECMO), consisting of a pump, membrane oxygenator, and heater. Depending on local laws, femoral vessels are cannulated pre-mortem or post-mortem (whereby inferior vena cava and abdominal aorta are also used). Heparin is administered accordingly. Following death and cannulation, deoxygenated blood is drained from the donor's venous system, and oxygenated blood is returned to the arterial circulation. Of note, a cross clamp across the descending thoracic aorta, or an endoluminal occlusion balloon within it, prevents perfusion of the brain, while a vent in the ascending aorta ensures that the brain is not perfused.

NRP is typically performed in the operating theatre or intensive care unit, with varying durations (1–4 h or longer in Italy).

### 3.2 *Ex situ* normothermic machine perfusion after organ retrieval

Unlike *in situ* regional perfusion, which is almost universally managed at normothermia, various *ex situ* machine perfusion modalities in organs from DCD donors and marginal organs from DBD donors (e.g., ECD organs) have been designed. In liver transplantation, two types are utilized in the clinical preservation: NMP and (dual) hypothermic oxygenated machine perfusion (HOPE/D-HOPE) (59). The benefits of NMP have been evaluated using two approaches: as end-ischemic NMP after initial SCS and as continuous NMP from the time of organ procurement (preservation NMP) (60). Notably, however, while NMP has shown advantages for liver (61), heart (62), lung (63), and kidney transplants (64), it cannot entirely prevent the effects IRI.

Here, we address only NMP that is applied in solid organ transplantation, mainly in liver and kidney transplantation [for recent reviews, see (65–69)]. Conceptually, NMP recapitulates near-physiological circulation, temperature, and cell metabolism, aimed at restoring function *ex vivo* and enhancing (extended) preservation of donor organs. With the use of this setup, metabolites, oxygen, targeted medication, and circulation are supplemented by continuous circulation of a warmed (35°C–38°C), oxygenated, red blood cell (RBC)-based, plasma-free perfusate fluid through the donor organ. The ultimate goal of this maneuver is to minimize postischemic reperfusion-mediated allograft injury, associated with a longer warm ischemia period (compare Part 1, Section 4.3.1).

Notably, NMP can be commenced during or immediately after procurement at the donor site, implicating, however, logistic hurtles requiring transport of a complex machine to the site of organ procurement in an peripheral donor hospital. Alternatively, the perfusion preservation procedure takes place at the recipient transplant center following the transportation of the donor organ under standard SCS [known as end-ischemic NMP, back-to-base NMP, or back-to-hub NMP (70)].

#### 3.2.1 Technique

In principle, the technical *ex situ* setup of NMP of the liver and the kidney includes an organ chamber, a centrifugal perfusion pump, an oxygenator, a heat exchanger, and monitoring devices to measure flow, pressure, and temperature [reviewed in (58, 71, 72)]. Specifically, during normothermic liver perfusion, the liver is supplied with oxygenated blood via both hepatic artery and portal vein, whereby the latter may either be via dependent flow or directly pumped. The effluent blood then flows back through a venous cannula and is pumped through the circulation again. There are two types of devices currently available for liver NMP, depending upon whether the perfusion circuit is open to air or closed [for details, see Watson et al. (58)].

For kidney NMP, a similar setup is used, adapted to the anatomical vessel situation of kidneys. In this case, normothermic perfusion serves as an emerging technique that uses cardiopulmonary bypass technology with extracorporeal membrane oxygenation to perfuse kidneys with a warmed and oxygenated red-cell-based plasma-free solution [for further reading, see Elliot et al. (71) and Hosgood et al. (73)].

The duration of NMP in liver and kidney transplantation varies, with periods for the liver ranging from 3 h to several days (usually 16 h) (58), and for kidneys ranging from 1 h to 24 h (71, 74). Excitingly, Clavien et al. (75) reported the successful transplantation of a human liver that could be even preserved for several days using *ex situ* NMP. Recognizing the significance of their findings, the authors anticipate the potential for extending the preservation period to up to 10 days of NMP in future applications. Of note, normothermic perfusion is most effective when applied during the entire period of organ preservation (76) while end-ischemic applications in marginal DBD and DCD livers failed to protect from the development of ischemic-type biliary lesions and subsequent graft loss (77).

#### 3.2.2 The downside of normothermic perfusion: reperfusion injury-induced, DAMP-driven inflammation and immunogenicity

Growing evidence, especially for end-ischemic NMP, indicates that normothermic perfusion preservation can induce undesirable IRI in the potential allograft (78). While it is generally assumed that NRP induces minimal, if any, IRI, there is a notable lack of robust data to substantiate this claim. Conversely, initial findings from studies on a clinically relevant rat DCD liver graft model have demonstrated that NMP can induce minimal to moderate IRI, contingent on the extent of initial donor warm ischemia, as reported by Schlegel et al. (79). These observations led the authors to discuss, among other considerations, how normothermic oxygenated perfusion activates the entire cascade of reperfusion injury, evidenced by the release of DAMPs, referencing prior work from our group (9, 80)*.* Subsequent experimental and clinical investigations have corroborated these earlier findings on NMP-induced IRI (81).

Of note, preliminary evidence for the emission of DAMPs has already emerged from clinical and experimental studies conducted in various *ex situ* perfusion preservation settings, including high mobility group protein B1 (HMGB1) (82, 83) and free heme from damaged RBCs (84, 85). The existing understanding of these two NMP-induced DAMPs that reflect the immunogenicity of the donor organ during NMP is certainly not the end of the story. It does not take a prophet to predict that further reports on the emission of DAMPs during NMP preservation will emerge soon.

Moreover, in alignment with contemporary inflammation research, emerging evidence suggests that NMP may induce both a DAMP-promoted initiation of an inflammatory response and a suppressive DAMP (SAMP)-driven resolution of inflammation [for further details, see (86)]. For example, in a careful clinical analysis of liver biopsies using single-cell transcriptome profiling of human donor livers prior to, during NMP and after transplantation, Hautz et al. (87) found several interleukins and chemokines released into the perfusate during NMP, including tumor necrosis factors (TNF) and IL-6, followed by a shift to anti-inflammatory markers such as the IL-10 cytokine (87). In view of this data, it is tempting to assume - as previously discussed for IRI in Part 1, Section 5.3.3 - that NMP-induced post-translational modifications may have converted the proinflammatory activity of specific DAMPs into anti-inflammatory/immunosuppressive effects.

Ultimately, however, it is crucial to emphasize that while NMP may cause mild to moderate reperfusion injury, its ability to significantly reduce post-transplant IRI in the recipient by effectively limiting hypoxic damage far outweighs this drawback. As a consequence of this effect, it significantly improves the function of marginal transplant organs, making it an indispensable strategy for optimizing transplant success.

#### 3.2.3 Advancing research on modern perfusion preservation techniques: ischemia-free normothermic perfusion of transplant organs

Overall, there is an increasing trend towards NMP becoming a widely accepted and commonly used method for preserving organs from marginal donors, such as ECD and DCD donors. This emergence of the NMP approach will create opportunities to utilize this technique for improved viability assessment and therapeutic interventions, but may also have a downside: inducing an unwanted adverse IRI. This can be explained by the fact that restoring normal cellular metabolism and replenishing cellular energy stores during perfusion enables the molecular pathways to drive RCD formation in conjunction with subsequent release of DAMPs. Therefore, therapeutic interventions aimed at controlling RCD and DAMPs must begin as early as possible – ideally, at the onset of NRP, or at the onset of NMP if NRP is not employed.

However, advancements in the normothermic perfusion of transplant organs are far from complete and continue to evolve. Thus, current research in this field is culminating in the development of ischemia-free normothermic perfusion technology. In fact, building on our original danger/injury hypothesis, which posits that IRI to a transplant organ initiates and amplifies innate alloimmune responses (1, 88, 89), He et al. (90, 91) recently introduced a novel protocol of ischemia-free normothermic perfusion of transplant organs in liver transplantation. Indeed, ischemia-free liver transplantation is a groundbreaking approach that ensures a continuous oxygenated blood supply to the liver of brain-dead donors throughout the entire process - from procurement and preservation to implantation - using NMP technology. This innovative approach, which also enables a real-time *ex vivo* assessment of liver function and viability, is currently swiftly earning widespread recognition and attention within the transplant community. In fact, a recent prospective, randomized controlled trial in patients with end-stage liver disease demonstrated that ischemia-free liver transplantation significantly reduces complications associated with IRI, when compared to conventional approaches (92). Remarkably, it has even been shown to fully prevent IRI (93). This advancement holds the potential to greatly improve transplant outcomes in the future. However, the question remains whether DAMPs released during prior injuries to donor organs, before the implementation of the ischemia-free protocol, may still have the capacity to activate both donor- and recipient-derived DCs.

### 3.3 Outlook

Overall, the current literature indicates that both NRP and NMP are viable strategies that have the potential to expand the donor pool by enhancing graft utilization, while maintaining acceptable transplant outcomes and likely achieving improved results compared to traditionally preserved DCD organs. Research in this field is going on as reflected by the recent development of a program of ischemia-free normothermic perfusion. The exploration and application of RCD and DAMPs as biomarkers and therapeutic targets, however, offers the potential to further refine these perfusion strategies, ultimately enhancing patient outcomes. Proposals for integrating these novel approaches are outlined in the following sections.

## 4 Leveraging RCD and DAMPs as biomarkers: advancing viability assessment in normothermic organ perfusion

Normothermic regional perfusion and NMP are advanced techniques primarily utilized to evaluate the quality and functionality of ECD transplant organs. In contrast to traditional cold storage preservation, these methods restore metabolic activity, enabling the assessment of donor organ dysfunction resulting from injury under near-physiological conditions, both *in situ* and *ex situ*, prior to transplantation. This policy facilitates real-time viability assessment during preservation by utilizing perfusion criteria and biochemical parameters. Recent reviews on the viability assessment of donor livers and kidneys during NRP (94) and NMP (95) have highlighted these advancements. Thus, for liver and kidney transplantation, markers like transaminase release, lactate metabolism, and bile production (liver) or perfusate flow, pH and *neutrophil gelatinase-associated lipocalin* levels (kidney) are key viability indicators [for further reading, see (58, 96–100)].

Overall, viability assessment protocols during normothermic perfusion of ECD transplant organs leveraging currently available biomarkers are evolving. Incorporating novel markers offers significant potential to refine these protocols and improve graft selection. As such valuable biomarkers, the role of RCD and DAMPs in NRP and NMP is briefly outlined below. Indeed, their integration into these organ preservation techniques is compellingly underscored by findings from studies on organ injury models and related diseases, which highlight their utility in enhancing the understanding and prediction of organ viability. A few examples are presented in the following.

### 4.1 RCD-related molecules as biomarkers in diseases

The determination of valuable biomarkers specific to forms of RCD represents an emerging diagnostic and prognostic tool in medicine, offering insights into the role of RCD in the pathology of human diseases. For example, molecular biomarkers used for the detection of necroptosis including RIPK1, RIPK3, and MLKL are already used clinically and have been shown to serve as early prognostic biomarkers in patients with sepsis and other critical illnesses (101–105).

Identification of biomarkers for pyroptosis is also emerging as a diagnostic approach for diseases. They include intracellular markers [such as caspases, granzyme, and members of the gasdermin (GSDM) family], and cell-released substances [such as interleukin (IL)-1β and IL-18] (106). For example, serum levels of GSDMD were proposed to serve as a potential predictive biomarker of prognosis in patients with end-stage renal disease (107) and IL-1β in patients with ulcerative colitis (108).

For ferroptosis, no biomarkers had been identified for a considerable time. However, recent studies have provided initial evidence suggesting a potential prognostic role for *SRY-box transcription factor 13* (SOX13) in ferroptosis within the context of thyroid cancer and for *proto-oncogene serine/threonine-protein kinase Pim-1* (PIM1) in ferroptosis associated with abdominal aortic aneurysm (109, 110). As noted by Zeng et al. (111), advancements in ferroptosis detection methods and their applications are expected to become prominent areas of research in the coming years.

The detection of NETosis currently relies on biomarkers such as citrullinated histones, the co-localization of neutrophil-derived proteins with extracellular DNA, and the presence of cell-free DNA (112). The citrullinated histone H3, for example, was found to be a predictor of ischemic stroke/transient ischemic attacks in patients with atrial fibrillation (113).

Actually, an emerging tool for detecting cell death involves measuring the membrane protein ninjurin-1(NINJ1), which enables the identification of not only a specific form of RCD but also various types of cell death characterized by plasma membrane rupture (PMR). For instance, circulating NINJ1 levels were shown to be elevated in septic patients and positively correlated with sepsis severity scores (114). Undoubtedly, these findings highlight the prospective clinical utility of NINJ1 as a promising versatile marker for detecting various types of RCD using just a single method during NRP and/or NMP (for NINJ1, see Part 1, Section 3.2.1 and Figure 6). Consequently, future rigorous validation studies will be crucial for its successful implementation in normothermic perfusion protocols for transplant organs.

### 4.2 DAMPs as biomarkers in diseases

Continuous circulation of perfusate in a static volume guarantees that DAMPs once released, remain within the system. The use of DAMPs as biomarkers during NRP and/or NMP would allow to predict the extent of tissue injury and transplant organ function more precisely as well as to provide first insights into DAMP-promoted autoinflammation. However, beyond this already valuable information, analyzing the DAMPs profile would also facilitate assessment of donor organ immunogenicity conferred by DAMP-activated donor DCs via direct allorecognition, which contributes to the initiation of alloimmunity.

To highlight the ability of DAMPs to provide such critical information, their role as reliable markers of acute injury to the kidney, liver, and lungs – as previously demonstrated in other settings - is briefly outlined here.

In clinical studies on patients (including pediatric patients) with acute kidney injury (AKI), DAMPs, specifically S100A8/S100A9 and urine mitochondrial DNA (mtDNA), have been identified as potential diagnostic and prognostic biomarkers, reflecting, for example, the severity of this disease (115–117). In similar investigations on critical ill surgical patients (118), elevated urinary mtDNA levels were able to diagnose newly developed AKI and predict renal replacement therapy or hospital mortality in those patients. Finally, the use of urinary mtDNA as a biomarker of renal dysfunction could be confirmed in patients with AKI following cardiac surgery (119).

Regarding acute liver injury, DAMPs such as HMGB1, nuclear DNA (nDNA), and mtDNA have already been used as biomarkers in acetaminophen-induced intoxication/hepatotoxicity and demonstrated a good correlation with the course of this acute disease (120). Additionally, as a notable finding of clinical studies, the determination of plasma levels of the DAMP HMGB1 surpassed all other biomarkers in predicting the clinical outcome of patients with acetaminophen overdose (121, 122). In this context, it is also worth noting a report on a significant elevation of plasma HMGB1 levels in patients with acute alcoholic hepatitis (123), which is assessed by the authors as a potential biomarker for improved prediction of disease progression and survival.

DAMPs have also been identified as promising biomarkers in acute lung injury (ALI) (124–127). For example, informative HMGB1 levels could be measured in bronchoalveolar lavage (BAL) fluid from trauma patients suffering from mechanical ventilator-associated pneumonia (126). Similarly, a study on patients with severe pneumonia and acute respiratory distress syndrome (ARDS) requiring mechanical ventilation (127) identified day-1 HMGB1 levels as a critical and independent biomarker for ICU mortality. Moreover, studies on ARDS patients revealed that extracellular histone levels in plasma and BAL fluid are significantly higher in ARDS patients than in controls (128). Clinical data analysis further demonstrated a strong association between elevated extracellular histones and increased ARDS severity and mortality. These observations are supported by a clinical study in patients with suspected ventilator-associated pneumonia, in which higher levels of mtDNA were detected in the BAL fluid (129).

### 4.3 RCD and DAMPs as biomarkers in normothermic perfusion of transplant organs

#### 4.3.1 RCD-related molecules

Notably, cell death-related molecules have already been proposed to use as potential biomarkers during *ex vivo* lung perfusion (130). Ideally, these molecules should be measured in perfusates at the beginning of NRP of EDC organs. This would provide an initial insight into the injuries the potential transplant organ was exposed to at different stages: (i) during events prior to ICU admission (e.g., shock episodes), (ii) during the ICU stay (e.g., cardiac arrest/resuscitation IRI), and (iii) under DCD conditions (compare Part 1, Section 4.3.2). However, measurement of RCD-related molecules could also be recommended in cases where NRP has been considered for future application to organs from DBD donors. Also in this case, this policy would provide first insights into those injuries the potential allograft was exposed to during events prior to ICU admission (e.g., shock events) and, during ICU stay, under DBD condition (e.g., brain-death-related inflammatory disturbance of peripheral organs, compare Part 1, Section 4.2.2). A targeted therapeutic intervention with RCD inhibitors can be precisely planned and carried out according to the results of this examination, for example, at the onset of NMP subsequently performed.

The rationale for measuring RCD-related molecules in NMP corresponds with that for NRP but offers the additional possibility of detecting injuries the transplant organ has sustained after removal during the preservation process (e.g., SCS).

#### 4.3.2 DAMPs

DAMPs such as HMGB1 and extracellular DNA have already been identified as useful biomarkers for assessment of the function of perfused organs in a previous clinical and experimental study (82, 83). Subsequently, it has also already been proposed to use mitochondrial DAMPs as biomarkers to assess the transplant suitability of procured DCD hearts and ultimately aid in facilitating the safe, widespread adoption of DCD heart transplantation (131). Moreover, a recent clinical study on *ex situ* NMP of EDC donor livers revealed that mtDNA levels in perfusate fluid and bile, as assessed by a real time PCR-based approach, correlate with donor liver quality (132). Certainly, the range of DAMPs to be measured in NRP and/or NMP for an appropriate evaluation of organ quality needs to be expanded in the near future to include cDAMPs and iDAMPs.

For practical application, the timing of measuring DAMPs as biomarkers is challenging. Taking into account the fact that commercially available assays such as ELISA kits require about 2–5 h time, DAMPs such as HMGB1, heat shock proteins (HSPs), S100 protein, calreticulin (CALR), eATP, and IFN-I should ideally be measured in perfusates as early as possible, e.g., at the beginning of NRP and/or NMP of EDC organs. This approach would offer immediate feedback for real-time clinical decision-making regarding the “transplantability” of an organ (e.g., discarding an organ for transplantation). On the other hand, measurement at the end of NMP would be helpful in assessing the overall extent of injury to the transplant organ in relation to its viability and function, including potential damage during perfusion. Apart from that, this timing would allow the measurement of nucleic acid DAMPs using the available rapid PCR methods.

### 4.4 Outlook

Collectively, the measurement of RCD-related molecules and DAMPs as biomarkers – especially DAMPs - obviously offers a substantial advancement in the methodologies currently used for viability assessment during normothermic perfusion techniques. For example, compared to biomarkers in liver NMP that merely assess end products such as bile production and lactate clearance, DAMPs provide valuable insights into earlier donor organ injuries. Moreover, DAMPs provide transplantologists with critical insights into the potential magnitude of the NMP-associated inflammatory response they promote, as well as the inherent immunogenicity of the donor organ that they determine. With multiple evaluation methods already yielding promising results, future efforts should prioritize integrating RCD-related molecules and DAMPs as biomarkers with traditional markers to enhance predictive accuracy and global applicability. However, currently available commercial assays, such as ELISA kits for measuring eligible cDAMPs and iDAMPs, may be insufficient and inadequate. Therefore, collaboration between the industry and clinical transplantologists will be essential to expand and refine the range of available diagnostic products in this field in the near future.

## 5 Preventing and inhibiting RCD and DAMPs during normothermic perfusion of transplant organs: pretransplant therapeutic options

The utilization of NRP and/or NMP for transplant organs helps sustain a level of aerobic metabolism, which supports metabolic activity and thus offers an opportunity to implement targeted therapies aimed at mitigating IRI in the recipient. Specifically, these therapeutic interventions could focus on reducing IRI-triggered innate alloimmune responses. Consequently, several research groups are currently exploring these technologies in preclinical studies as platforms for delivering novel therapeutics to recondition, repair, and optimize organs for transplantation, including efforts to reduce immunogenicity (133–137).

Notably, a conceptual strategy to suppress injury-induced innate alloimmune events in organ transplantation during the time of normothermic organ preservation was initially proposed by us in 2006 (138), and later in 2011/2012 (45, 139). This approach considered several therapeutic options, including the use of pharmaceutics, biologics, and gene therapy (RNA interference). Additionally, to prevent activation of immunostimulatory DCs, potential therapeutic strategies were ordered according to different levels of DAMP-triggered, PRR-mediated signaling pathways in DCs, taking the following steps: DAMP inhibitors [e.g., monoclonal antibodies (mAbs) against HMGB1] → PRR inhibitors → transcription factor inhibitors. Some of these early suggestions are now being implemented in preclinical models of NMP of organs from ECD donors, including approaches such as monoclonal antibody (mAb) therapy (140), gene therapy (141), and cell therapy (142). Collectively, these emerging technologies in therapeutic interventions during NMP show significant promise for improving organ transplantation outcomes in the future.

Here, however, the focus is on the role of RCD and RCD-derived DAMPs as therapeutic targets in NRP and/or NMP. In fact, the rationale for inhibiting, blocking, or removing DAMPs that have accumulated from all previous periodic donor organ injuries is to prevent, after allograft implantation, activation of both residual donor-derived and graft-infiltrating recipient-derived PRR-bearing innate immune cells, including DCs, which otherwise results in alloinflammation and alloimmunity.

On the other hand, as a prophylactic intervention, therapeutic targeting of RCD types aims to prevent the release of a new wave of DAMPs from these potentially dying cells during subsequent IRI. While some degree of this oxidative injury may eventually occur during NRP or NMP, it is vigorously pronounced during allograft reperfusion following implantation. Furthermore, independent of targeting RCD→DAMPs, a new approach proposes targeting costimulatory molecules on donor-derived DCs that have already been activated by DAMPs, with the goal of preventing or mitigating the process of direct allorecognition.

### 5.1 Strategies for inhibiting regulated cell death

A valuable addition to therapeutic interventions such as those mentioned above - particularly for reducing the immunogenicity of organs from ECD donors – involves the inhibition of RCD pathways. The rationale behind this approach is that forms of RCD not only serve as prolific sources of cDAMPs, but they also indirectly promote the secretion of iDAMPs by innate immune cells activated by cDAMPs. Consequently, types of RCD that would arise in the donor organ during phases of IRI – i.e., during NMP and subsequently after implantation in the recipient - must be prevented during NRP or NMP by adding RCD agonistic inhibitors (antagonists) to the perfusate. Reasonably, this approach can be considered an effective strategy for drastically reducing the emission of cDAMPs and iDAMPs during IRI of the donor organ after implantation into the recipient. Several therapeutic interventions targeting distinct types of RCD (ferroptosis, necroposis, pyroptosis, NETosis) are currently available and will be briefly discussed below. Notably, however, a novel strategy has been recently reported that applies a single mAb, which may simultaneously inhibit multiple forms of RCD. Specifically, the inhibition of NINJ1 oligomerization – which is implicated in PMR across several forms of RCD (see Part 1, Section 3.2.1) - using a mAb has provided evidence suggesting that it can prevent the ultimate execution of necroptosis, pyroptosis, and (at least partially, see below) ferroptosis (143, 144). Exploring the effect of NINJ1 blockade in the setting of NMP, where the release of DAMPs from RCD subsequently during IRI post-implantation should be prevented, represents an exciting avenue for future research. However, as noted by Kayagaki et al. (143), it “*will require reagents with improved pharmacokinetic properties*”.

#### 5.1.1 Ferroptosis inhibitors

The concept of targeted treatment of ferroptosis in human diseases is currently a prominent area of research. In fact, earlier studies have already proposed the therapeutic inhibition of ferroptosis in conditions such as IRI and related diseases (145), during the perioperative period of cardiac transplantation (146), and even in the context of *ex vivo* liver cold storage (147).

Originally, a first ferroptosis inhibitor (termed 16–86) to protect against IRI was already described in 2014 (148). In the meantime, numerous strategies have been explored to disrupt ferroptosis at various stages, demonstrating the growing interest in targeting this pathway [for reviews, see (149, 150)]. Remarkably, even a virally encoded protein, vPIF-1, has been identified for its potent antiferroptotic properties, indicating ferroptosis to potentially function as a viral defense mechanism (151).

Here, we briefly mention four classes of preclinically established ferroptosis inhibitors (ferrostatins) that are comprehensively discussed by Maremonti et al. (144). The first class of ferrostatins comprises iron chelators that inhibit lipid peroxidation. However, some clinical trials using iron chelators failed to provide clear protective effects e.g., in AKI (152). Class 2 ferrostatins refers to radical trapping agents that function in the cytoplasmic compartment. Some compounds have entered clinical routine such as omeprazole, rifampicin, and propranolol (153). Class 3 consists of lipophilic radical-trapping antioxidants (RTAs), which are by far the most extensively studied category of ferrostatins. The growing number of lipophilic RTAs is too extensive to enumerate here. A promising compound is liproxstatin-1 (Lip-1) that protected GPX4-deficient mice from death by acute renal tubular necrosis and therefore is considered a particularly suitable ferrostatin for *in vivo* research (154). The metabolite 7-dehydrocholesterol (7-DHC) out of this class is also regarded as a potential therapeutic agent (155). Class 4 ferrostatins are inhibitors that prevent the catastrophic burst of the plasma membrane. Currently, the only available example is the anti-NINJ1 mAbs (144), and it is likely that interfering with NINJ1 acts as a mechanism for inhibiting ferroptosis.

Overall, while the side effect profile of ferrostatins appears to be minimal, no clinical trials have been published to date. Furthermore, as previously noted, the design of such trials is challenged by the absence of suitable biomarkers for detecting ferroptosis in serum samples or tissue biopsies. These challenges underscore the importance of exploring the potential of promising ferrostatins in the NMP of transplant organs.

#### 5.1.2 Necroptosis inhibitors

Within the necroptosis pathway, RIPK1, RIPK3, and MLKL are now broadly acknowledged as pivotal therapeutic targets (for detailed reviews, see (156–160). Thus, targeting these molecules of the necroptotic signaling pathway presents a promising approach to inhibiting necroptosis in the contexts of NRP and/or NMP.

Inhibitors of RIPK1 were the first inhibitors of necroptosis discovered. They include necrostatins [e.g., necrostatin 1 (Nec-1)], dihydropyrazoles, benzoxazepinones, and some others such as type I and II kinase inhibitors of RIPK1. Various inhibitors in animal models such as the small molecule inhibitor Nec-1f (161) have been shown to protect against inflammation and cell damage and IRI. A few RIPK1 inhibitors have been evaluated in patients with various diseases, most inhibitors were well tolerated, however, no efficacy has been reported for these RIPK1 inhibitors, but many studies are still ongoing. Inhibitors of RIPK3 include compounds such as amino benzothiazole compounds, BMS compounds, and some other RIPK3 inhibitors such as ponatinib, dabrafenib, and sorafenib. Small molecules targeting MLKL include necrosulfonamide, xanthine-based ligands, other small molecules targeting the N-terminal domain of MLKL such as uracil compounds, and aminopyrimidines. However, it should be noted that most of the studies dealing with therapeutics targeting necroptosis are grounded on *in vitro* experiments and/or animal models. Therefore, the clinical effectiveness of these necroptosis inhibitors still needs to be evaluated by clinical control trials. Given that RCD is currently a hot topic in biomedicine, valid data from clinical trials on the efficacy of various inhibitors is anticipated in the near future. In this context, the question arises as to whether inhibition of the PMR in necroptosis, in conjunction with ferroptosis and pyroptosis, is more effectively achieved through NINJ1 inhibition (143).

#### 5.1.3 Pyroptosis inhibitors

In recent years, there has been growing interest in the development of drugs targeting the inhibition of pyroptosis. This has led to the identification and progression of NLRP3 inhibitors with various chemotypes, and early clinical trials are currently assessing the safety and efficacy of the most promising candidate therapies. Furthermore, the inflammasome effector GSDMD has garnered significant attention due to its potential as a comprehensive therapeutic target, given its essential role in multiple inflammasome pathways [for reviews, see (162–166)]. For example, *the cytokine release inhibitory drugs* (CRIDs) were shown to inhibit IL-1β release with nanomolar potency. Thus, CRID3, one of the CRID molecules, has been demonstrated to inhibit NLRP3 inflammasome activation in both human and murine cells with nanomolar potency (167). Due to its exceptional selectivity and potency, CRID3 continues to serve as the gold standard tool compound for inhibiting NLRP3 in preclinical disease models (164). Other small molecule NLRP3 inhibitors such as ZYIL1 (168) and GDC-2394 (169) and DFV890 (170) are in clinical development, but as oral formulations. Likewise, GSDMD inhibitors have demonstrated promising results in mouse models of inflammatory diseases, and a number of new inhibitors are being identified [reviewed in (165)]. Current strategies to inhibit GSDMD mainly focus on binding to GSDMD, preventing its cleavage, or inhibiting the oligomerization of its N-terminal (NT) region, although these approaches may have some off-target effects. Several compounds using these strategies are currently in clinical development. For example, FDA-approved disulfiram was found to inhibit pyroptosis by blocking GSDMD pore formation (171). Moreover, a retrospective cohort study has demonstrated that disulfiram reduces the incidence and severity of COVID-19. The compound is currently being assessed in two Phase II clinical trials (NCT04485130 and NCT04594343) for the treatment of COVID-19 (172). As proposed by Lucas-Ruiz et al. (173), targeting GSDMD inhibition could improve therapeutic effectiveness in NMP by blocking pyroptosis across all inflammasome pathways. Notably, to our knowledge, no pyroptotic inhibitors have been clinically developed for intravenous administration so far.

#### 5.1.4 NETosis inhibitors

The formation of NETs (NETosis) is another type of RCD induced by IRI to allografts (174, 175). Several anti-NETs therapeutics have been described including *protein arginine deiminases* (PAD) inhibitors such as chlor-amidine and hydroxychloroquine, calcineurin inhibitors such as ciclosporin, treatment with DNase such as recombinant human DNase1, and ROS scavengers [for reviews see (176, 177)]. Most important with respect to inhibition of NET formation during NMP is the use of special extracorporeal blood purification (EBP) devices, which are able to remove NETs (178) (see also below).

#### 5.1.5 Targeting NINJ1

As said, there is currently an exciting discussion on the possibility that all types of RCD defined by PMR can be inhibited with the use of an antibody against NINJ1 (143). In fact, regulated cell lysis was originally considered a passive process that follows pore formation. However, as already mentioned in Part 1, Section 3.2.1, the integral plasma membrane protein NINJ1 has recently been discovered to have a crucial role in PMR during forms of RCD, establishing membrane rupture as a tightly regulated, active process. Consequently, NINJ1 actively drives the extracellular release of DAMPs such as HMGB1 that are too large to pass through pores as, for example, caused by GSDMD in pyroptosis. Given that NINJ1 might turn out to be a key mediator of PMR, targeting NINJ1 might present the ideal therapeutic approach to be used during NMP.

### 5.2 Therapeutic strategies for controlling DAMPs

Targeting DAMPs therapeutically to prevent IRI-promoted alloinflammation and alloimmunity is not a new idea. Our early proposal to therapeutically target DAMPs during normothermic organ preservation to prevent DC activation (139), for example, has recently been revisited by the Oxford group, who suggested the removal of DAMPs during liver NMP to facilitate organ rescue and reconditioning during preservation. As impressively proposed by the researchers (179), removal of NETs - containing DAMPs such as histones and cell-free DNA- via a special EBP device is achievable during NMP by connecting an apheresis device to the NMP device.

That said, the topic is likely broader: it involves controlling all those DAMPs – i.e., cDAMPs and iDAMPs - accumulated in the donor organ and identified as biomarkers, which - following allograft implantation - cause alloinflammation and - via DC activation - trigger an alloimmune response (compare Figure 2). Since there is evidence that DAMPs are generated in transplant organs not only during reperfusion after their implantation but also during SCS (180) and NMP (83), strategies to inactivate these danger-signaling molecules should already be implemented as early as possible – ideally - as also suggested by Lucas-Ruiz et al. (180) - during NRP, if performed at all, or at the onset of NMP (for cDAMPs and iDAMPs known to activate DCs, see Part 1, Section 5.4.3). To achieve this goal, various therapeutic options are available that are mainly based on results from *in vitro* experiments and preclinical studies to control DAMPs – here exemplified by some selected studies on DAMP-suppressing strategies in models of IRI and other disorders.

#### 5.2.1 Targeting DAMPs in ischemia/reperfusion injury and other disorders

Several therapeutic strategies are available for controlling DAMPs in diseases where they drive dysregulated innate immune responses. These options include EBP devices (ab-/adsorption), small molecule inhibitors/antagonists, enzymatic degradation, and mAbs (86). However, among these therapeutic options, EBP devices hold particular significance due to their widespread clinical application in ICUs for organ support and removal of toxin or DAMPs as discussed for sepsis (181). In contrast, while preclinical studies have demonstrated the effectiveness of specific inhibitors or mAbs targeting DAMPs, their routine clinical implementation is still pending. To stay within the allotted scope, only a few selected DAMPs will be discussed in the following.

##### 5.2.1.1 HMGB1, HSPs, and S100 proteins

HMGB1, a prototypic DAMP, has been extensively studied for its therapeutic potential in controlling sterile inflammation including IRI, sepsis, autoimmune diseases and cancer (182). Of great advantage in view of intervening with this DAMP during NMP is the fact that circulating HMGB1 can be efficiently removed by plasma adsorption membranes (183). Indeed, as reviewed (184), preclinical studies have consistently demonstrated that various hemofilters effectively eliminate HMGB1, primarily through adsorption technique, thereby mitigating DAMP-mediated immunopathological effects. For the future, the application of antibodies or inhibitors is equally relevant. For example, in studies on IRI models in rodents, treatment with an anti-HMGB1 mAb was found to reduce HMGB1 plasma levels and significantly improve the severity of IRI-related pathologies (185–187). Similarly, an HMGB1 neutralizing chimeric antibody was shown to attenuate drug-induced liver injury and postinjury inflammation in mice (188). In regard to HMGB1 inhibitors, glycyrrhizin, a naturally occurring compound that binds to extracellular HMGB1, was recently reported to attenuate myocardial IRI by suppressing inflammation, oxidative stress, and ferroptosis (189). Moreover, therapeutically targeting HMGB1 exerts a much broader impact on innate immune processes: As reviewed (190), early interventions with anti-HMGB1 antibodies or HMGB1 inhibitors have been shown to effectively reduce local and systemic inflammatory responses, mitigate T cell depletion, and alleviate organ failure.

Extracellular heat shock proteins such as HSP70 have also been shown to be released during IRI (191, 192) and are involved in IRI to transplant organs (193). The development of HSP inhibitors is under way. As reviewed (194), the development of HSP90 inhibitors is the most advanced, while other HSPs, including HSP70, gp96, and HSP27, are being researched as vaccines or antisense oligonucleotides.

The induction of S100 proteins such as S100A8/A9, during conditions of IRI, as observed in cardiovascular diseases (CVD) and allograft reperfusion, is well established (195, 196). For therapeutic intervention with S100 proteins, preclinically tested S100 neutralizing antibodies and small molecule inhibitors are available, which can be used to control the progression and severity of diseases. For instance, studies in a murine lung IRI model revealed that treatment with anti-S100A8/A9 mAb significantly reduced plasma levels of S100A8/A9 (197). As evaluated by oxygenation capacity and neutrophil infiltration, the antibody treatment - similar to antibody treatment of HMGB1- drastically improved IRI. Of interest is also the pharmacological inhibitor of S100A8/A9 *paquinimod*. In studies on a murine inflammatory model, this compound was shown to significantly reduce the accumulation of inflammatory monocytes and eosinophils during sterile inflammation (198). Remarkably, paquinimod treatment was also observed to lead to almost 100% survival in a lethal model of mouse coronavirus infection using the mouse hepatitis virus (199).

##### 5.2.1.2 Extracellular nucleic acids and histones

Cell-free DNA, particularly mtDNA (200–204), extracellular RNA (205, 206), and histones (207) are released from dying cells during IRI conditions, including in transplant organs, positioning them as valuable therapeutic targets in NRP and/or NMP. For example, apart from the removal of mtDNA via an EBP device (179), experimental studies in rats could demonstrate that neutralizing circulating mtDNA (and nDNA) using the nucleic acid scavenging polymer *hexadimethrine bromide* (HDMBr) was associated with significant recovery from severe multiple organ injury (208). Furthermore, studies in mice have shown that the removal of cell-free DNA by deoxyribonuclease-I (DNase-I) has neuroprotective effects when administered in the early phase, 1 day after traumatic brain injury (209). Moreover, in studies on a rat model of intestinal IRI associated with detectable levels of extracellular DNA in the serum, treatment with DNase-I was observed to reduce the inflammatory response (210).

Cell-free RNA represents another potential target for mitigating IRI to allografts (205). However, this area of research is less advanced compared to DNA scavengers. Theoretically, potential approaches could include RNA scavenging polymers, RNA-binding proteins, RNA-degrading enzymes, tailored nanoparticles, or antisense oligonucleotides.

Other key DAMPs involved in IRI refer to histones (207, 211). As recently reviewed by Yang et al. (212), the latest advances in preclinical studies on histone-targeting therapies include approaches such as heparin administration, anti-histone antibodies, histone-binding proteins or molecules, and histone-affinity hemoadsorption techniques. For example, recent studies have highlighted the potential of hemadsorption techniques in reducing circulating histone levels. Thus, a six-hour hemoadsorption procedure using a sophisticated adsorption device was shown to significantly reduce histone levels in the blood of multiply injured humans, with *in vitro* results showing 92%–99% adsorption efficiency (213). In a subsequent study, another specialized extracorporeal hemoadsorption device was shown to also effectively eliminate histones in septic plasma samples (214).

##### 5.2.1.3 Heme

Heme is a special DAMP that is not released from a type of RCD but from RBC due to membrane disruption during storage and NMP (84, 85). Heme is known to activate cells of the innate immune system and has emerged as a critical modulator of inflammatory responses and immune cell functions (215–217). Scavenging of heme by human serum albumin was shown to provide protection against free heme oxidative damage and was proposed to use for reducing inflammation, including IRI-associated inflammation (84, 218).

#### 5.2.2 Targeting DC-activating DAMPs

Most critical for the suppression of allograft rejection is the therapeutic targeting of those DAMPs that have been identified as key drivers of DC activation needed to initiate an alloimmune response [discussed above and in Refs (8–11, 13, 20).]. While the full spectrum of DAMPs with this specific capability is not known, initial insights into their existence have emerged from studies on immunogenic cell death (ICD) investigating the role of DCs in initiating antitumor immune responses. In fact, the cDAMPs HMGB1, HSPs, eATP, CALR, and the iDAMPs type-I IFNs have been identified as pivotal DAMPs that drive antitumor immune responses through the activation of DCs (219). Conversely, these critical DAMPs should be carefully controlled during NRP and/or NMP to avoid DC activation. Additionally, other DAMPs known to activate DCs must also be considered as therapeutic targets, including components of the extracellular matrix (ECM) such as hyaluronan (220) and heparan sulfate (221), as well as nucleic acids like mtDNA (222–227). Undoubtedly, this list is not exhaustive, and further future research in the field of normothermic perfusion preservation is needed to identify additional DAMPs: a truly challenging task for the next generation of transplant researchers!

Potential therapeutic strategies to control some of these DAMPs have been briefly outlined above. For other cDAMPs such as eATP, CALR, and hyaluronan, therapeutic options have also been addressed and discussed. For example, experimental studies using a mouse model of systemic inflammation have demonstrated that removing eATP with systemic apyrase treatment is an effective strategy for reducing systemic inflammatory damage and toxicity (228). In line with this approach, drugs modulating the eATP concentration in the perfusate should be a new frontier in therapeutic interventions during normothermic transplant organ perfusion. For inhibition of CALR - should this DAMP be found to be upregulated during IRI -, mAbs are available (229).

Finally, DC-activating iDAMPs such as type I IFNs and TNF can be targeted pharmacologically in a variety of ways. Indeed, potent type I IFN biologics are in clinical development (230, 231), while TNF biologics are routinely used in clinical practice (232).

### 5.3 The adjunctive therapeutic approach: blockade of costimulatory molecules on DAMP-activated donor dendritic cells

Even if activation of DCs by DAMPs during IRI to the allograft could be completely prevented through prior elimination of RCD and DAMPs during NMP, there remains a theoretical possibility that donor-derived DCs, previously activated by DAMPs during earlier periods of donor organ injury, could still mediate the initiation of an alloimmune response via direct allorecognition. Indeed, it is important to realize that direct allorecognition triggers a polyclonal alloimmune response engaging 1%–10% of the T cell repertoire, which represents a significanly high frequency of T cell activation (37).

With respect to the ambitious goal of minimizing the alloimmune response or even inducing allograft tolerance, it is essential to target the inactivation of these donor DCs in addition to deactivating/eliminating RCD and DAMPs. This can be accomplished by blocking the costimulatory molecules that are upregulated on the surface of these DCs, thereby inhibiting their ability to activate T cells (compare Part 1, Section 5.4.2). Indeed, blockade of costimulatory molecules can induce tolDCs (233) and has been shown to induce allograft tolerance in preclinical rodent models (234, 235). However, the goal of achieving long-term allograft tolerance in the clinic remains elusive.

Promising results from preclinical studies have already led to the clinical development of several inhibitors targeting costimulatory molecules on DCs, with ongoing studies focusing on autoimmune diseases and organ transplantation [for further reading, see (19, 236–238)]. For example, an FDA-approved drug for post-renal transplant immunosuppression is the CTLA4Ig fusion protein belatacept that binds CD80/86 (B7-1/B7-2) molecules (239). Two additional fusion proteins XPro952348 and MEDI5256 have a greater binding affinity to CD80/86 (240). Beyond the CD80/86↔CD28 costimulatory pathways, other therapeutics targeting the CD40↔CD154 costimulatory pathway are also under clinical development. For example, an antagonistic anti-CD40 mAb, bleselumab, has been evaluated in a phase IIa study involving kidney transplant recipients (241), another anti-CD40 antibody, iscalimab, has undergone a phase IIb trial in patients with Sjögren's disease (242). Moreover, a fully human antibody targeting ICOSL, AMG 557, was investigated in a phase Ib study in patients with systemic lupus erythematosus (243). Additionally, a fully human anti-OX40l mAb, amlitelimab, was tested in a phase IIa trial for patients with atopic dermatitis (244).

### 5.4 Outlook

The idea of selecting RCD and DAMPs as therapeutic targets – alongside the blockade of costimulatory molecules on DCs- in normothermic perfusion of transplant organs represents a logical progression in general medical-scientific thinking. It integrates two emerging concepts into the ever-evolving field of transplantology, with the overarching goal of enhancing outcomes in organ transplantation. This therapeutic concept is anticipated to align with the advancement of other innovative treatments that have become central to transplant research in NRP and NMP. Beginning with pre-clinical perfusion models, we envision this approach advancing toward initial clinical studies as its acceptance continues to grow. Certainly, unforeseen challenges and obstacles are to be expected, and it is unlikely that all objectives will be fully achieved in this endeavor. For instance, additional forms of RCD, such as parthanatos and cuproptosis, may also contribute to the release of DAMPs and will require intervention through the use of small-molecule inhibitors [for details see also (245)]. Nonetheless, this innovative strategy is expected to at least alleviate the burden of immunosuppressive therapy to some extent. Thus, it holds promise for addressing the significant comorbidities linked to current immunosuppressants, such as heightened risks of viral infections and malignancies.

## 6 The ambitious goal of leveraging normothermic perfusion of transplant organs for the induction of allotolerance

### 6.1 The dream of allotolerance induction

As briefly sketched in the prologue of Part 1, the focus of our considerations in this work is on the ambitious and visionary goal of leveraging RCD and associated release of DAMPs as therapeutic targets in NRP and NMP, with the aim of not only profoundly suppressing innate alloimmune responses, but even inducing allograft tolerance. Indeed, induction of allotolerance remains the aspirational dream of transplant researchers and clinicians over 70 years: Attaining a state of allograft-specific unresponsiveness would minimize reliance on lifelong daily immunosuppressive medications, improve post-transplant quality of life, and lead to superior long-term transplant outcomes (10, 246, 247).

Of note, the increasing application and advancement of NRP and NMP technology in solid organ transplantation, coupled with the advancing therapeutic strategies to prevent RCD and inhibit released DAMPs, provides a unique opportunity not only to minimize allograft dysfunction as linked to IRI-induced alloinflammation, but also bring us closer to fulfilling the “allotolerance dream”.

To reiterate and more precisely, the challenging objective of therapeutic interventions during NRP and/or NMP is to prevent the release of DAMPs (cDAMPs and iDAMPs), or – if they are present - to inhibit or remove them. The purpose of this strategy is to hinder these molecules from activating PRR-bearing innate immune cells - both those resident in the donor organ and those infiltrating from the recipient - after the organ has been implanted into the recipient. Successfully achieving this is anticipated to significantly reduce inflammation in the allograft post-implantation, thereby enhancing allograft function.

Beyond this endeavour, building on the discussion in Section 2.1.4, the broader goal is to present alloantigens by DCs in the absence of DAMPs to promote T cell tolerance in the recipient. Two potential approaches to achieve this goal can be considered:

(i) Promotion of homeostatic DC maturation: This strategy aims to exclude activation of residual PRR-bearing donor DCs and infiltrating recipient DCs by DAMPs, thereby enabling these APCs to adopt tolerogenic properties;
(ii) Administration of donor HLA antigens prior to transplantation: This maneuver takes advantage of the extended preservation time enabled by prolonged NMP to administer non-immunogenic doses of donor HLA antigens to the recipient prior to allograft implantation.

### 6.2 Promotion of homeostatic DC maturation in the absence of DAMPs

**Figure 3:**
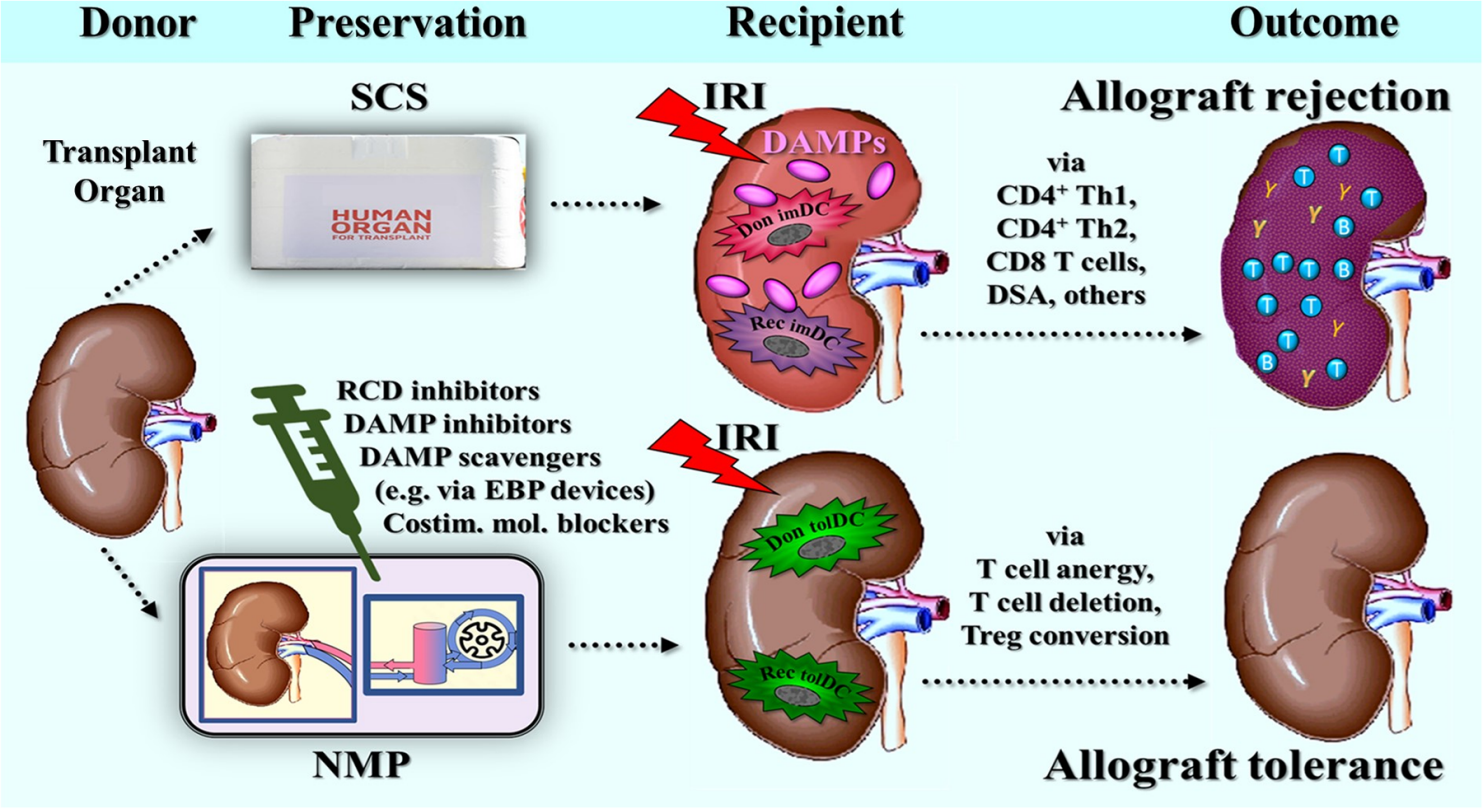
Schematic presentation of the hypothesis that successful inhibition and/or inactivation of intragraft RCD and DAMPs during normothermic perfusion of transplant organs, here exemplified by normothermic machine perfusion (NMP), promotes allograft tolerance. Below in the figure: The hypothesis posits that during postischemic reperfusion of the donor organ in the recipient, the therapeutically achieved absence of intragraft DAMPs – under steady-state -like conditions – enables the homeostatic maturation of both donor- and recipient-derived dendritic cells (DCs) into tolerogenic DCs (tolDCs). These tolDCs promote allograft tolerance via T cell anergy, T cell deletion, and T cell conversion into regulatory T cells. Top of the figure: In contrast, in the presence of injury (mainly postischemic reperfusion injury) - induced DAMPs, if no interventions have been initiated to inhibit or disable them, donor and recipient DCs mature to immunogenic antigen-presenting cells that interact with naïve T cells to initiate and amplify an alloimmune response. for injuries to the allograft, see Part 1, Chaps. 4 and 5; also compare above Figures 1, 2. DSA, donor-specific antibodies; Costim. mol., costimulatory molecules; Don imDC, donor-derived immunogenic dendritic cell; Don tolDC, donor-derived tolerogenic dendritic cell; EBP, extracorporeal blood purification; Rec imDC, recipient-derived immunogenic dendritic cell; Rec tolDC, recipient-derived tolerogenic dendritic cell; SCS, static cold storage; Treg, regulatory T cells.

Given the above delineated scenario of the divergent function of DCs in allorecognition, preventing DAMP-driven immunogenic maturation of DCs while simultaneously promoting homeostatic DC maturation is the key to inducing successful allotolerance. In other words, the therapeutic interventions during NRP and/or NMP, aimed at preventing DAMP release through RCD inhibition and inhibiting and disabling DAMPs already present in the transplant organ, are hypothesized to establish a steady state-like environment within the allograft, thereby preventing alloimmune activation and promoting allograft tolerance (Figure 3). To implement this approach during normothermic perfusion of organ transplants, several critical actions must be undertaken and addressed. Thus, RCD inhibitors should be applied to the perfusion system as early as possible, that is, at the beginning of NRP from deceased donors before organ retrieval, with the aim to prevent release of DAMPs during subsequent preservation phases (e.g., SCS or NMP) and reperfusion after implantation. Together with the application of RCD inhibitors, blockers of costimulatory molecules must be added to inactivate donor-derived DCs already activated by DAMPs during previous injuries to the donor organ. If the NRP procedure has not been performed, administration of RCD inhibitors should be scheduled for the very beginning of NMP of the transplant organ to prevent release of DAMPs during ongoing NMP and subsequent reperfusion in the recipient (Figure 3).

From a stringent transplantological standpoint, aiming to achieve allograft tolerance in all patients irrespective of logistical and financial constraints, this approach should be applied not only to DCD donors but also to DBD donors.

Executing these actions effectively is undeniably fraught with challenges and obstacles. A key question is whether it is feasible to completely inhibit or eliminate all those DAMPs in the donor organ able to activate PRR-bearing cells of the innate immune system. In this regard, the Oxford group's project represents a promising starting point (179), but the number of DAMPs removed by their methods is presumably insufficient. Also, the above mentioned list of DAMPs proposed as potential therapeutic targets is incomplete and likely does not cover all DAMPs expressed in injured donor organs. This raises a critical question: are these therapeutic efforts overly ambitious or even illusory, given there are too many DAMPs that would need to be effectively controlled?

In this context, a stepwise approach appears rational for future preclinical and clinical studies focused on identifying DAMPs and developing therapeutic strategies to target them during NRP and/or NMP. This process would involve parallel collaborating with the pharmaceutical industry to develop novel assays for measuring distinct DAMPs and to create innovative therapeutic strategies to inhibit or remove these molecules, along with testing potential agonistic inhibitors, scavengers, or EBP techniques.

In particular, this research should prioritize the development of innovative EBP techniques, such as hemoadsorption devices, designed to target and remove the widest possible range of DAMPs [see (248)!]. Additionally, research efforts should focus on preparing advanced oligoclonal antibody cocktails or recombinant polyclonal antibodies against a broad spectrum of DAMPs, with the goal of administering these therapies to the perfusate during NRP or NMP [see (190)]. The efficacy of these emerging therapeutic interventions, implemented during NRP and/or NMP, can be evaluated in preclinical models or clinical trials by examining their correlation with subsequent clinical outcomes, including the extent of IRI-associated allograft dysfunction and the incidence of acute rejection episodes.

### 6.3 Administration of donor HLA antigens to the recipient before transplantation

With a prolonged use of NMP over several days as reported (75), another opportunity arises to induce allotolerance that differs from leveraging RCD and DAMPs as therapeutic targets: the administration of soluble donor HLA antigens to the recipient prior to allotransplantation. Indeed, without the pressure of time, purification of soluble HLA antigens from donor leukocytes and their subsequent application to recipients as alloantigens in a non-immunogenic context should become a viable option in the future. The concept is again rooted in the danger/injury paradigm positing that presentation of antigens in the absence of DAMP-elicited costimulatory signals fosters the induction of antigen-specific immunological tolerance. This principle also applies to autoantigens and is not a novel idea in immunology.

#### 6.3.1 Administration of autoantigen in the absence of DAMPs

Indeed, earlier autoantigen-specific strategies to block autoimmune responses using peptides or whole antigens have evolved into more targeted approaches. These efforts now aim to deliver the autoantigenic molecules to autoreactive T cells either directly via MHC molecules (e.g., soluble peptide/MHC) or indirectly, via APCs such as tolDCs, more recently through DNA or mRNA vaccines, in a way intended to drive clonal deletion and/or immunoregulation. These various antigen-specific therapeutic approaches for autoimmunity have been comprehensively reviewed by Serra and Santamaria (249), by Robinson and Thomas in the context of systemic lupus erythematosus (250), and more recently by Song et al. (251). In relation to allotolerance, this principle was also reviewed by us (46). The concept also applies to autoantigens involved in multiple sclerosis (MS). In fact, approaches to induce antigen-specific tolerance by administering putative autoantigens pathogenetically implicated in MS have already been undertaken, using designs that have shown promise in experimental studies. However, as pointed out and discussed by Serra and Santamaria (249), given the still not crystal-clearly identified putative autoantigens, peptide-based or soluble peptide/MHC-based clinical interventions for this disease have been largely unsuccessful.

A new approach in this field is the use of a noninflammatory mRNA vaccine, as published by Krienke et al. (252). The concept here is that systemic administration of nanoparticle-formulated N1- methylpseudouridine-modified messenger RNA encoding putative autoantigens in MS leads to antigen presentation on APCs in the absence of DAMPs, that is, absence of costimulatory signals. In studies on various MS models in mice, the researchers found the disease to be suppressed with the use of such nanoparticle/modified mRNA. The authors further demonstrated that the treatment effect is associated with a reduction in effector T cells and the development of Tregs. Of note, the authors found that these Tregs exert a strong bystander immunosuppression and thus alleviate the disease triggered by cognate and noncognate autoantigens. However, as Radbruch and Melchers (253) also recently discussed, the approach of active vaccination with autoantigens using mRNA technology still faces significant challenges. To achieve the ultimate goals of permanently suppressing established unwanted immune responses and restoring immunological tolerance, “ there is still a long way to go”.

#### 6.3.2 Administration of alloantigen (allopeptide) in the absence of DAMPs

In organ transplantation, this concept of autoantigen-based therapy would imply inducing successful allotolerance by administration of antigens in the absence of injury/DAMPs. The idea here would be to administer purified soluble donor alloantigens (i.e., mismatched HLA antigens, e.g., via MHC molecules) to the recipient prior to transplantation, that is, during the time when the transplant organ is under NMP. In fact, one advantage over autoantigens here is that alloantigens are more precisely defined. Accordingly, experiments should be envisaged and designed to allow the presentation of such antigens under non-immunogenic conditions in an undamaged noninflammatory microenvironment to recipients. Of note, this concept has already been successfully applied in a murine model (254): Female mice infused with a single class II MHC-presented HY peptide via osmotic minipumps developed Treg-mediated long-term tolerance to all male-specific HY transplantation antigens.

Clinically, based on emerging therapeutic strategies to induce antigen-specific immune tolerance for the treatment of autoimmune diseases [reviewed in (251)], approaches to deliver non-immunogenic alloantigen preparations may include oral administration or nasal inhalation, or intravenous delivery in the form of [lipid] nanoparticle-based alloantigens, respectively.

A rather attractive approach for clinical application in transplant recipients appears to be the *peptide-MHC-based nanomedicine* concept proposed by Serra and Santamaria (249). The therapeutic strategy would involve engineered nanoparticles coated with multiple copies of donor pMHC molecules. In their experiments with various nanoparticle types, the researchers (249) demonstrated - besides others - that it is not the absolute number of pMHC monomers per nanoparticle that determines potency, but rather the density of these pMHCs on the nanoparticle surface, “*such that NPs of different sizes carrying identical numbers of pMHCs will have different potencies*”.

Future advancements in nanoparticle and pMHC engineering principles are eagerly anticipated, as they could potentially lead to a transformative progress in the potential revolutionization of therapeutic strategies for inducing allotolerance.

The hypothetically envisioned culmination of such an experimental approach would be pre-transplant intravenous administration to the recipient of nanoparticle-formulated N1 methylpseudouridine-modified mRNA encoding the desired mismatched donor MHC/HLA antigens. This experimental → clinical approach would parallel studies on several mouse models of MS, which showed that such treatment results in antigen presentation on APCs in the absence of costimulatory signals (252, 255). Of note, at least theoretically, this approach could also be applied to recipients of xenografts from genetically modified pigs (“xenotolerance” induction) independently of the use of NRP/NMP. In this case, porcine soluble xenoantigens [swine leukocyte antigens (256)] would be used - if these are well defined in the future.

Future experiments will show us whether these considerations on alloantigen-specific induction of allotolerance remain purely theoretical or hold real potential for practical application. When probatorily evaluating these abovementioned two innovative approaches to inducing allograft tolerance we are inclined to favor the mRNA-based technology. This method generates mRNA-encoded donor MHC/HLA antigens directly from the recipient's own cells, ensuring a natural process free from molecular disruptions and, thus, “free from DAMPs” - potentially even mimicking endogenous self-antigens for enhanced compatibility and tolerability.

### 6.4 Outlook

For years, transplantologists have been working on ways to promote allograft-specific immune tolerance. The goal is to reduce the need for high doses and multiple immunosuppressive drugs, giving the immune system a better chance to fight off infections and cancer. The two primary strategies for inducing transplant tolerance have been: (i) establishing a state of mixed chimerism through the transfer of donor hematopoietic stem cells to the recipient, thereby promoting central tolerance to alloantigens, and (ii) delivering alloantigens to the recipient in a non-immunogenic fashion to activate peripheral tolerance mechanisms toward the allograft. The question is of whether or not we could come closer to our desired goal when applying the concept described here. Given the many dissappointments in reaching this goal in the past, one has to be very cautious in being too optimistic.

## 7 Epilogue

This bipartite review, which explores strategies used during normothermic perfusion techniques to mitigate or even disable the recipient's immune defense against a foreign donor organ, should be concluded with a brief holistic perspective on the evolutionary foundations of host defense mechanisms. Indeed, the role of the bio-entity *RCD*→*DAMPs* in alloimmune-mediated transplant rejection - as discussed in this review - can be considered just a “tiny particle of the major universal whole shaped by evolutionary processes”. Thus, modern notions in evolutionary research hold that all living organisms on our planet rely on DAMPs for their daily defense against all kinds of injuries, whether caused by sterile conditions or infections – an ongoing “struggle for life”, as highlighted in “*DAMPs across the Tree of Life*” (257).

Accordingly, the scenarios outlined in these two parts of the review can also be seen from an evolutionary perspective as an inherent intertwining of RCD and DAMPs in terms of a highly conserved mechanistic tool for host defense against injury. As described in Part 1, Chap. 3, all the diverse types of RCD, each induced by activators (or “stressors”) triggering distinct molecular trajectories, converge on the same endpoint: the loss of membrane integrity and rupture of the plasma membrane. Remarkably, it is these fundamental biological momenta that serve as the primary source for the release of both iDAMPs (through pores) and cDAMPs (through the ruptured plasma membrane). In other words: the induction of regulated necrosis is an indispensable part of DAMP release and vice versa!

**Figure 4:**
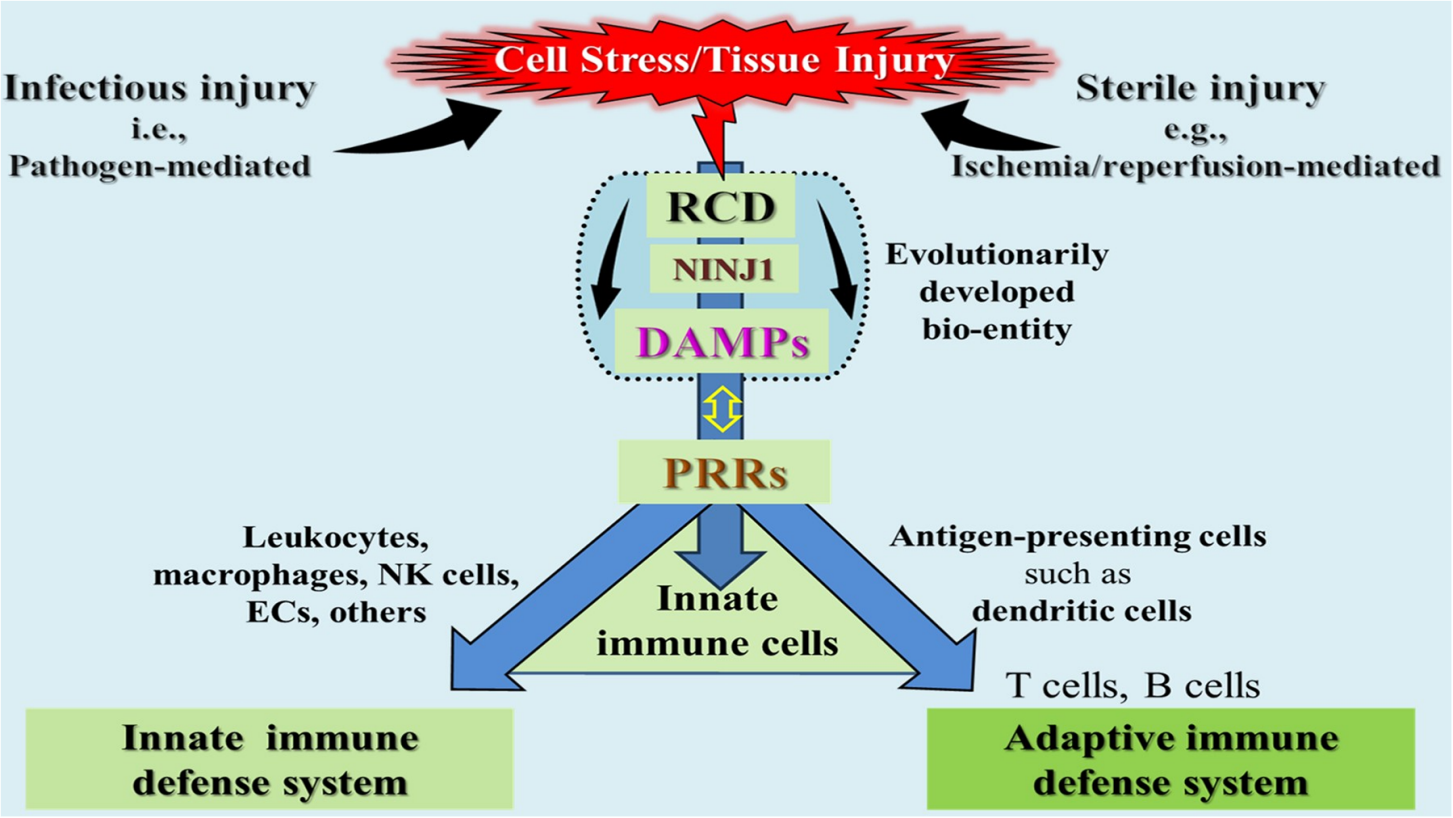
A schematic representation of the hypothetical model of the evolutionarily developed bio-entity “*RCD*→*DAMPs*”, illustrating its role as a critical initial component of the evolutionar*y* axis in host defense mechanisms against any severe injuries, whether induced by harmful sterile events or invading pathogens. Further evolutionarily highly conserved components contributing to the execution of host defense in this context include the membrane protein NINJ1 that controls the release of DAMPs from RCD, and pattern recognition receptors (PRRs) on/in innate immune cells, which become activated after interacting with DAMPs. Activated cells of the innate immune system such as leukocytes, macrophages, natural killer cells (NKs), and endothelial cells (ECs) constitute the innate immune defense system. In parallel, antigen-presenting cells such as dendritic cells promote the activation of T cells, thereby driving the molecular and cellular processes constituting the adaptive immune defense system. NINJ1, ninjurin-1; PRRs, pattern recognition receptors; RCD, regulated cell death.

When seeking an explanation for this unique, inherent intertwining of RCD and DAMPs, one should consider Theodosius Dobzhansky's assertion: “Nothing in biology makes sense except in the light of evolution” (258). Indeed, viewed through the lens of evolution, the following hypothetical model of an evolutionar*y* axis in host defense against any stress and/or injury across the tree of life can be proposed (Figure 4): Any severe infectious or sterile stress/damage to an organism that cannot be homeostatically managed by cell-autonomous stress responses leads to a type of RCD. The different signaling pathways involved in the different types of RCD - viewed as essential evolutionarily conserved trajectories to restore homeostasis following injury (259, 260) - appear to be adjusted to the nature of a given insult and ultimately lead to the active process of PMR via the action of NINJ1. The molecule NINJ1 is again a highly conserved plasma membrane resident protein in mammals and is widely expressed in various tissues and cell types (261). Finally, NINJ1-driven PMR actively allows release of DAMPs, which again are highly conserved, evolution-dictated defense molecules across the tree of life, dedicated to repairing and regenerating tissue following tissue injury (257). Engagement of DAMPs with highly conserved PRRs expressed on/in cells of the innate immune system including APCs, then, initiates and amplifies innate/adaptive immune defense responses.

Overall, it is reasonable to conclude that this fundamental, highly conserved inherently intertwined bio-entity of RCD→DAMPs has evolved as part of a powerful evolutionarl*y* axis of defense against any form of severe cell stress and/or tissue injury. In the context of allograft injury, the recipient's defense system responds according to its evolutionary mission: to protect the host from perceived threats. However, this response contrasts with the transplant surgeon's intentional goal of alleviating the recipient's suffering through transplantation. In this sense, allograft rejection can be considered the result of a fateful confusion by the immune defense system of a beneficial intervention and a dangerous threat. But in evolution's defense, it really could not have anticipated that “puzzle people” (262) would one day in the future be transplanting organs.
